# Supplementary material for: Important features of retail shoes for women with rheumatoid arthritis: A Delphi consensus survey
Source: PLoS One. 2019 Dec 27;14(12):e0226906. doi: 10.1371/journal.pone.0226906 (PMC6934318; doi:10.1371/journal.pone.0226906)
Supplement: S1 Table — (DOCX) [file pone.0226906.s001.docx]

**Supporting Information**

**S1 Table: Results of all Rounds from Delphi Survey**

|  | | | | | | | | |
| --- | --- | --- | --- | --- | --- | --- | --- | --- |
| **Round 1: Open (collated responses)** | | | | | | | | |
| A shoe for a woman with RA should:  Provide adequate shock absorption  Should contain features found in trainers/running shoes  Be activity appropriate  Be made of breathable material  be made from soft fabric  be suitable for the weather conditions (warm in winter, cool in summer)  be lightweight  be a boot style if additional support is required  A shoe for a woman with RA should:  Be slip on  have Velcro fastenings  be lace up  be well secured  be easy to put on and take off  have fastenings which are adjustable  Have an outsole which is textured and non-slip  Have a thick outsole  Have a soft accommodative upper  Should have a leather upper  Should have an upper which is conformable  Should contain an insole which is removable  Should contain a supportive inlay  Contain adequate midfoot support  Have longitudinal arch support  Have a wide toe box  Have a deep toe box  Contain metatarsal support  Contain forefoot to rearfoot cushioning  Have a rocker in metatarsal areas  Be adjustable in metatarsal areas  Have a round toe box  Be able to accommodate toes  Contain a semi-rigid shank  Have a sturdy rearfoot  A heel height of no greater than 3 cm (1.5 inches)  A cushioned heel  Contain a heel stability cup  Have a broad heel base  Have a stiff heel counter  Should not contain a sling back  Should contain a heel counter which conforms to the geometry of the calcaneus  Shoe comfort should be preferable to aesthetic  Shoes should be aesthetically pleasing  Shoes should be appropriately sized, with adequate length and width  Women with RA should be professionally fitted for their footwear  Shoes should be deep enough to accommodate an orthoses/insert  Shoes should not have seams over pressure areas or deformities  Shoes should avoid pressure points in the foot  Shoe shape should be relevant to foot shape  Women should own two pairs of shoes which can be alternated | | | | | | | | |
| **Round 2: results from panel** | | | | | | | | |
| *Accepted* | *Median (30-70 IQR)^A^* | *DI^A^* | *Rejected* | *Median (30-70 IQR)^A^* | *DI^A^* | *Ambiguous* | *Median (30-70 IQR)^A^* | *DI^A^* |
| Shoes should be appropriately sized, with adequate length and width | 9 (9-9) | 0 | Shoes should be one size larger than required | 2.0 (2-4) | 0.29 | Contain metatarsal support | 6.0 (6-7.8) | 0.46 |
| Shoes should be deep enough to accommodate an orthoses/insert | 8 (7-8.8) | 0.26 | be slip on | 3.0 (2-5) | 0.29 | Have longitudinal arch support | 6.0 (6-7) | 0.25 |
| Shoes should not have seams over pressure areas or deformities | 9 (7.2 -9) | 0.21 |  |  |  | Women with RA should be professionally fitted for their footwear | 6.0 (5-7) | 0.51 |
| Shoes should avoid pressure points in the foot | 9 (8-9) | 0.11 |  |  |  | Women should own two pairs of shoes which can be alternated | 5.0 (4 – 7.8) | 1.62 |
| Shoe shape should be relevant to foot shape | 8 (7-9) | 0.29 |  |  |  | Shoe comfort should be preferable to aesthetic | 6.0 (5-7) | 0.51 |
| Shoes should be aesthetically pleasing | 7 (7-8) | 0.18 |  |  |  | Contain a heel stability cup | 6.0 (5-7) | 0.51 |
| There should be a range of choice in styles available | 8 (7-8.8) | 0.26 |  |  |  | Have a stiff heel counter | 6.0 (5-7) | 0.51 |
| There should be a range of choice in colours available | 8 (7-8) | 0.14 |  |  |  | Should not contain a sling back | 6.0 (5-8) | 0.89 |
| Shoes should be deemed satisfactory by the owner | 9 (8-9) | 0.11 |  |  |  | Have a rocker in metatarsal areas | 6.0 (5-6) | 0.25 |
| Shoes should look feminine | 7 (6-7) | 0.18 |  |  |  | Be adjustable in metatarsal areas | 6.0 (6-7.8) | 0.25 |
| Shoes should not cause self-consciousness | 8 (8-9) | 0.14 |  |  |  | Have a round toe box | 6 (5-6.8) | 0.25 |
| Shoes should be appropriate for the social occasion | 7 (6.2 – 8) | 0.33 |  |  |  | Contain a semi-rigid shank | 6 (5-7) | 0.25 |
| Have a sturdy rearfoot | 7 (6-8) | 0.37 |  |  |  | Have a thick outsole | 5 (5-7) | 0 |
| A heel height of no greater than 3 cm (1.5 inches) | 7 (6-8) | 0.37 |  |  |  | Should have a leather upper | 5 (4-6) | 0 |
| A cushioned heel | 7 (6-7) | 0.18 |  |  |  | Should contain a supportive inlay | 6 (5.2-7) | 0.25 |
| Have a broad heel base | 7 (6-8) | 0.37 |  |  |  | have Velcro fastenings | 5 (4-6) | 0 |
| Should contain a heel counter which conforms to the geometry of the calcaneus | 7 (5-8) | 0.56 |  |  |  | be lace up | 5 (3.2 -6) | 0 |
| Contain adequate midfoot support | 7 (6.2-8) | 0.39 |  |  |  | contain features found in trainers/running shoes | 6 (5-7) | 0.25 |
| Have a wide toe box | 8 (7-8) | 0.89 |  |  |  | be made from soft fabric | 6 (5-7) | 0.25 |
| Have a deep toe box | 7 (6-8) | 0.37 |  |  |  | be a boot style if additional support is required | 6 (5.2-7) | 0.25 |
| Contain forefoot to rearfoot cushioning | 7 (6-7.1) | 0.5 |  |  |  |  |  |  |
| Be able to accommodate toes | 8 (8-9) | 0.77 |  |  |  |  |  |  |
| Have an outsole which is textured and non-slip | 8 (7-8) | 0.89 |  |  |  |  |  |  |
| Have a soft accommodative upper | 7 (6-8) | 0.37 |  |  |  |  |  |  |
| Should have an upper which is conformable | 8 (8-9) | 0.77 |  |  |  |  |  |  |
| Should contain an insole which is removable | 8 (6-9) | 0.43 |  |  |  |  |  |  |
| be well secured | 8 (7-9) | 0.37 |  |  |  |  |  |  |
| be easy to put on and take off | 8 (8-9) | 0.77 |  |  |  |  |  |  |
| have fastenings which are adjustable | 8 (6-9) | 0.43 |  |  |  |  |  |  |
| provide adequate shock absorption | 8 (6-8) | 0.56 |  |  |  |  |  |  |
| be activity appropriate | 8 (8-9) | 0.77 |  |  |  |  |  |  |
| be made of breathable material | 7 (5.2-8) | 0.30 |  |  |  |  |  |  |
| be suitable for the weather conditions (warm in winter, cool in summer) | 8 (7-9) | 0.37 |  |  |  |  |  |  |
| be lightweight | 7 (6-8) | 0.37 |  |  |  |  |  |  |
| be deemed comfortable by the owner | 9 (9-9) | 0 |  |  |  |  |  |  |
| be affordable | 9 (8-9) | 0.11 |  |  |  |  |  |  |
| ^A^A disagreement index of less than 1 indicates no disagreement | | |  |  |  |  |  |  |
| **Round 3: results from panel** | | | | | | | | |
| *Accepted* | *Median (30-70 IQR)^A^* | *DI^A^* | *Rejected* | *Median (30-70 IQR)^A^* | *DI^A^* | *Ambiguous* | *Median (30-70 IQR)^A^* | *DI^A^* |
| Contain metatarsal support | 7.0 (6-7) | 0.18 | Nil |  |  | Women with RA should be professionally fitted for their footwear | 6.0 (6-7) | 0.25 |
| Have longitudinal arch support | 7.0 (6-7) | 0.18 |  |  |  | Women should own two pairs of shoes which can be alternated | 5.5 (5-7) | 0.64 |
|  |  |  |  |  |  | Shoe comfort should be preferable to aesthetic | 6.0 (5-7) | 0.25 |
|  |  |  |  |  |  | Contain a heel stability cup | 6.0 (6-7) | 0.25 |
|  |  |  |  |  |  | Have a stiff heel counter | 6.0 (6-7) | 0.25 |
|  |  |  |  |  |  | Should not contain a sling back | 6.0 (5-7) | 0.25 |
|  |  |  |  |  |  | Have a rocker in metatarsal areas | 6.0 (5-6) | 0.25 |
|  |  |  |  |  |  | Be adjustable in metatarsal areas | 6.0 (6-7) | 0.25 |
|  |  |  |  |  |  | Have a round toe box | 6.0 (6-7) | 0.25 |
|  |  |  |  |  |  | Contain a semi-rigid shank | 6.0 (6-6) | 0 |
|  |  |  |  |  |  | Have a thick outsole | 6.0 (5-6) | 0.25 |
|  |  |  |  |  |  | Should have a leather upper | 5.0 (4-5) | 0.42 |
|  |  |  |  |  |  | Should contain a supportive inlay | 6.0 (6-7) | 0.25 |
|  |  |  |  |  |  | have Velcro fastenings | 5.0 (5-5) | 0 |
|  |  |  |  |  |  | be lace up | 5.0 (4-5) | 0.42 |
|  |  |  |  |  |  | contain features found in trainers/running shoes | 6.0 (6-7) | 0.25 |
|  |  |  |  |  |  | be made from soft fabric | 6.0 (5-6) | 0.25 |
|  |  |  |  |  |  | be a boot style if additional support is required | 6.0 (5-6) | 0.25 |
|  | | | | | | | | |

^A^A disagreement index of less than 1 indicates no disagreement
