# Supplementary material for: Important features of retail shoes for women with rheumatoid arthritis: A Delphi consensus survey
Source: PLoS One. 2019 Dec 27;14(12):e0226906. doi: 10.1371/journal.pone.0226906 (PMC6934318; doi:10.1371/journal.pone.0226906)
Supplement: Attachment — Submitted filename: responsetoreviewers.docx [file pone.0226906.s002.docx]

Reviewer #1: This Delphi study about features of retail shoes for women with rheumatoid arthritis is very important and strongly needed. The study is well designed consisted experts worldwide. Couple of minor points to revise:

-Background is very short. Can you consider broadening it to take into account footwear adherence and related factors.

*Thank you this has been added below:*

Therapeutic (medical grade or custom made) footwear is often prescribed for women with RA, however is frequently unavailable to many women with RA due to cost [6]. Furthermore, therapeutic footwear is often not accepted by women, which reduces adherence and therefore the opportunity to achieve therapeutic benefit is lost [7]. Lack of adherence with therapeutic footwear is multi-factorial, often compounded by a lack of acceptance of their underlying condition, and a disparity in expectations of the footwear [3, 7, 8]. This non-acceptance of therapeutic footwear results in many women seeking retail footwear as an alternative.

-Dot is missing before the aim of the study at the end of Background.

*Thank you this has been amended*

-Through the text you separate the UK from Europe, to my knowledge UK is part of Europe.

*Thank you this has been amended*

Moreover, in text and Table 1 location is based on coutry or continent - can you unify these? Maybe report countries where the experts came?

*Thank you this has been amended we have unified as continent to be consistent with the manuscript.*

-Maybe one recommendation could also be for footwear manufacturer? And inclusion of the to future research projects.

*Thank you this has been added: Furthermore, footwear manufacturers should take note of specific features which were considered important for women with RA and integrate these into future footwear designs.*

Thank you, very good study and well written.

Reviewer #2: Thank you for the opportunity to review this manuscript. It is of interest to researchers and health professionals who provide foot care and advice for people who have RA.

My comments are minor and primarily relating to some of the grammar only.
Abstract - research is performed with participants rather than on participants. Please consider modifying to:
An international Delphi exercise using online survey software was conducted with 39 participants from health care backgrounds.

*This has been amended thank you*

Also, there are multiple sentences that are missing full stops. Please reread and correct.

*This has been amended thank you*

Introduction:
Consider amending for plural consistency: whilst offering the woman a choice in style
To: whilst offering women choices in style

*This has been amended thank you*

Methods:
Amending: A sample of health professionals was
To: A sample of health professionals were

*This has been amended thank you*

Expertise - based on your inclusion criteria - I would suggest that the expertise was footwear prescription rather than just footwear?

*Thank you this has been amended*

At times in the methods section there are tense issues and would urge the authors to use past tense throughout

*Thank you this has been amended*

Results:
female with mean (SD) of 23 (10) mean years - delete duplicate mean

*Thank you this has been amended*

I note that the methods describe presenting the 30th and 70th percentile, IPA and then IQR? Please present one of the other or describe why these have been used in each section.

*Thank you for identifying this error – the 30-70^th^ interpercentile ranges were calculated and this has been amended within the text.*
